# Using Phenomenological Models to Characterize Transmissibility and Forecast Patterns and Final Burden of Zika Epidemics

**DOI:** 10.1371/currents.outbreaks.f14b2217c902f453d9320a43a35b9583

**Published:** 2016-05-31

**Authors:** Gerardo Chowell, Doracelly Hincapie-Palacio, Juan Ospina, Bruce Pell, Amna Tariq, Sushma Dahal, Seyed Moghadas, Alexandra Smirnova, Lone Simonsen, Cécile Viboud

**Affiliations:** Division of Epidemiology and Population Studies, Fogarty International Center, National Institutes of Health, Bethesda, Maryland, USA; Mathematical, Computational & Modeling Sciences Center, School of Human Evolution and Social Change, Arizona State University, Tempe, Arizona, USA; Logic and Computation Group, EAFIT University, Medellin, Antioquia, Colombia; School of Public Health, Georgia State University, Atlanta, Georgia, USA; School of Public Health, Georgia State University, Atlanta, Georgia, USA; Agent Based Modelling Laboratory, York University, Toronto, Canada; Department of Mathematics and Statistics, Georgia State University, Atlanta, Georgia, USA; Department of Public Health, University of Copenhagen, Copenhagen, Denmark; Division of International Epidemiology and Population Studies, Fogarty International Center, National Institutes of Health, Bethesda, Maryland, USA

## Abstract

Background: The World Health Organization declared the ongoing Zika virus (ZIKV) epidemic in the Americas a Public Health Emergency of International Concern on February 1, 2016. ZIKV disease in humans is characterized by a “dengue-like” syndrome including febrile illness and rash. However, ZIKV infection in early pregnancy has been associated with severe birth defects, including microcephaly and other developmental issues. Mechanistic models of disease transmission can be used to forecast trajectories and likely disease burden but are currently hampered by substantial uncertainty on the epidemiology of the disease (e.g., the role of asymptomatic transmission, generation interval, incubation period, and key drivers). When insight is limited, phenomenological models provide a starting point for estimation of key transmission parameters, such as the reproduction number, and forecasts of epidemic impact.

Methods: We obtained daily counts of suspected Zika cases by date of symptoms onset from the Secretary of Health of Antioquia, Colombia during January-April 2016. We calibrated the generalized Richards model, a phenomenological model that accommodates a variety of early exponential and sub-exponential growth kinetics, against the early epidemic trajectory and generated predictions of epidemic size. The reproduction number was estimated by applying the renewal equation to incident cases simulated from the fitted generalized-growth model and assuming gamma or exponentially-distributed generation intervals derived from the literature. We estimated the reproduction number for an increasing duration of the epidemic growth phase.

Results: The reproduction number rapidly declined from 10.3 (95% CI: 8.3, 12.4) in the first disease generation to 2.2 (95% CI: 1.9, 2.8) in the second disease generation, assuming a gamma-distributed generation interval with the mean of 14 days and standard deviation of 2 days. The generalized-Richards model outperformed the logistic growth model and provided forecasts within 22% of the actual epidemic size based on an assessment 30 days into the epidemic, with the epidemic peaking on day 36.

Conclusion: Phenomenological models represent promising tools to generate early forecasts of epidemic impact particularly in the context of substantial uncertainty in epidemiological parameters. Our findings underscore the need to treat the reproduction number as a dynamic quantity even during the early growth phase, and emphasize the sensitivity of reproduction number estimates to assumptions on the generation interval distribution.

## Introduction

The Zika virus (ZIKV) is an arbovirus that belongs to the family Flaviviridae and genus Flavivirus^1^. ZIKV was first isolated from a rhesus monkey in the Zika forest of Uganda in 1947^1^ . ZIKV is related to the dengue virus and is primarily transmitted to humans by Aedes aegypti bites, the mosquito currently predominantly implicated in transmission of ZIKV. Sexual transmission of ZIKV has also been reported^2^, and was first suspected in southeastern Senegal in 2008^3^, but the contribution of the different transmission pathways (mosquito bite vs. sexual contacts) to the overall force of infection of ZIKV dynamics remains unknown. In the United States a total of 426 imported cases have been reported in travelers returning from Zika-affected areas, while a total of 8 cases have been attributed to sexual transmission as of 30 April 2016 2

ZIKV disease in humans is characterized by a “dengue-like” syndrome, which consists of fever, rashes, conjunctivitis, arthralgia, myalgia, headache, and malaise. While human infections are usually asymptomatic or mild with self- limiting disease, resembling influenza-like illness^4^, ZIKV infection among pregnant women is of particular concern as perinatal infection has been associated with severe neurologic conditions including microcephaly and Guillain–Barré syndrome (GBS) 2 ^,^ 5 ^,^ 6 ^,^ 7.

The first human infection by ZIKV was reported from East Africa in 1952^8^ and was followed by sporadic cases in Asia and Africa during the 1960s^9^. ZIKV outbreaks were not reported until April 2007 in the Yap Island, Federated States of Micronesia and in the North Pacific^4^. These outbreaks were followed by a major outbreak consisting of about 28,000 reported cases in French Polynesia, South Pacific in October 2013^10^. In the Western Hemisphere, active circulation of ZIKV was first reported in Brazil in May 2015, which lead the Pan American Health Organization (PAHO) to issue an epidemiological alert. Active transmission of ZIKV is currently reported in 35 countries in the Americas since 2015^11^, and WHO declared the epidemic a Public Health Emergency of International Concern on February 1, 2016. Recent phylogenetic analyses indicate that the epidemic in the Americas was triggered by an imported case sometime between May and December 2013, a period that coincides with an increase in air travel from ZIKV affected areas in the Pacific to Brazil^12^.

Substantial uncertainty on the epidemiology of ZIKV (e.g., the role of asymptomatic transmission, the length of the incubation period and the generation interval) and the contribution of different modes of transmission (mosquito bites vs. sexual transmission) hinders the development of fully mechanistic models of disease transmission dynamics. In this context, phenomenological models provide a starting point for forecasting epidemic impact (e.g., epidemic size) and characterizing the temporal changes in the reproduction number during the early growth phase. Here we employ simple phenomenological models based on a few parameters, and assumptions about the serial interval, to analyze the reproduction number of Zika for the recent epidemic in Antioquia, Colombia, and generate early predictions of the epidemic size.

## Materials and Methods

**Data**

We obtained daily counts of suspected Zika cases by date of symptoms onset reported to the Secretary of Health of Antioquia. Antioquia is the second largest department in Colombia (with a population size of ~ 6.3 million people), located in the central northwestern part of the country. The epidemic peaked 36 days into the outbreak and consists of about 104 epidemic days as of 10 April 2016. On October 14th, 2015, The Colombia Ministry of Health issued detailed guidance to carry out epidemiological surveillance for Zika and confirmed the presence of the virus in the country on October 16, 2015. by December 2015, ZIKV was already circulating in 150 municipalities in Colombia. The Ministry of Health of Colombia has reported a total of 75,187 suspected cases of ZIKV, of which about 5% has been confirmed through laboratory tests as of 23 April 2016. The definition of a suspected case is broad^13^ while laboratory confirmation is based on PCR.

**Methods**

The reproduction number was estimated by applying the renewal equation to case incidence data simulated from the fitted generalized-growth model (GGM) and assuming gamma and exponentially-distributed generation intervals derived from the literature. For forecasting the epidemic in Antioquia, Colombia, we calibrated a generalized-Richards model (GRM), a phenomenological model that accommodates a variety of exponential and sub-exponential growth kinetics of the early epidemic trajectory, and generated predictions of the epidemic size. For comparison purposes, we also calibrated the logistic growth model to the epidemic data.

Simple epidemic models based on a small number of parameters have the potential to provide rapid epidemic forecasts and estimates of key transmission parameters based on the early trajectory of an outbreak 14 ^,^ 15 ^,^ 16 ^,^ 17 ^,^ 18 ^,^ 19. Here we employed the GRM developed based on the original Richards model 20 ^,^ 21 and the recently introduced generalized-growth model (GGM, \begin{equation*}C'(t)=rC(t)^{p}\end{equation*}) that incorporates a ‘deceleration of growth’ parameter (\begin{equation*}p\end{equation*} )^22^. Hence, we model the rate of change in the number of new cases at day t, \begin{equation*}C'(t)\end{equation*} , using a single differential equation:

\begin{equation*}C'(t)=rC(t)^p[1-(C(t)/K)^a]\end{equation*}

where \begin{equation*}r\end{equation*} represents the intrinsic growth rate in the absence of any control or saturation of disease spread, \begin{equation*}K\end{equation*} is the final size of the epidemic, \begin{equation*}a\end{equation*} is a parameter that modulates the peak-time of incidence^20^. Here, \begin{equation*}p\end{equation*} is a ‘deceleration of growth’ parameter ranges between 0 and 1, and modulates the early growth kinetics of the epidemic and can accommodate profiles ranging from constant incidence (\begin{equation*}p=0\end{equation*} ), polynomial growth (\begin{equation*}0<p<1\end{equation*} ), to exponential growth (\begin{equation*}p=1\end{equation*} )^22^ .

**Reproduction number**

We analyzed the reproduction number by calibrating the generalized-growth model (GGM)^22^ to an increasing length of the early growth phase comprising 30, 35, and 40 epidemic days, respectively 23 , and using information about the distribution of the generation interval for ZIKV reported in ref. 24 . We estimated the growth rate parameter \begin{equation*}r\end{equation*} and the deceleration of growth parameter \begin{equation*}p\end{equation*} in the GGM model as previously described 22. Next, we simulated the progression of incident cases from the calibrated GGM model, and applied the discretized probability distribution of the generation interval using the renewal equation 25 ^,^ 26 :

\begin{equation*}R(t_i)=\frac{I_i}{\sum_{j=0}^{i}{I_{i-j}\rho_j} } \end{equation*}

where \begin{equation*}I_i\end{equation*} denotes incidence at time \begin{equation*}t_i\end{equation*} , \begin{equation*}\rho_j\end{equation*} denotes the discretised probability distribution of the generation interval, and the denominator represents the total number of cases that contribute (as primary cases) to generating new cases \begin{equation*}I_i\end{equation*} (as secondary cases)^25^. Given the substantial uncertainty around the generation interval of ZIKV, we assumed a gamma distributed generation interval with a mean of 14 days and standard deviation of 2 days based on limited data reported in ref^24^. To assess the sensitivity of our estimates of the reproduction number to changes in the shape of the generation interval distribution, we also evaluated the reproduction number using an exponentially-distributed generation interval with a mean of 14 days.

**Epidemic forecasting**

We calibrated the GRM to daily Zika case incidence data for increasingly longer epidemic windows, from 20, 30, 40, 50, 60 and up to 70 days into the epidemic, respectively, corresponding to end dates ranging from 17 January 2016 to 07 April 2016. Then for each of these data inputs, we projected the model forward. For comparison purposes, we also employed the logistic growth model with two parameters \begin{equation*}r\end{equation*} and \begin{equation*}K\end{equation*} (where \begin{equation*}p=1,a=1\end{equation*} in the GRM model) to generate epidemic forecasts using this simpler model form. Estimates of model parameters were obtained by nonlinear least-squares curve fitting using the Levenberg-Marquardt algorithm in MATLAB (The Mathworks, Inc.)^22^. The initial number of cases was fixed according to the observed incidence data on 28 December 2015. Parameter uncertainty was quantified by simulation of 200 stochastic realizations of the best-fit curves using parametric bootstrap 27, with a Poisson error structure. The simulated epidemic curves were used to derive the nominal 95% confidence intervals for each parameter.

## Results

The trajectory of the ZIKV epidemic in Antioquia, Colombia, is displayed in Figure 1; most cases are concentrated during January-March 2015. Our estimates of the reproduction number using an increasing length of the early growth phase of the Zika epidemic displayed a rapidly declining trend from 10.3 (95% CI: 8.3, 12.4) in the first disease generation (i.e., 14 days into the epidemic) to 2.2 (95% CI: 1.9, 2.8) in the second disease generation (e.g., 28 days into the epidemic), assuming a gamma distributed generation interval with a mean of 14 days (SD=2) (Figure 2). When the generation interval distribution was assumed to be exponentially-distributed with a mean of 14 days, the reproduction number declined from 2.8 (95% CI: 2.4, 3.1) in the first disease generation to 1.8 (95% CI: 1.7, 2.0) in the second disease generation (Figure 3).

**Zika case series, Antioquia, Colombia:**
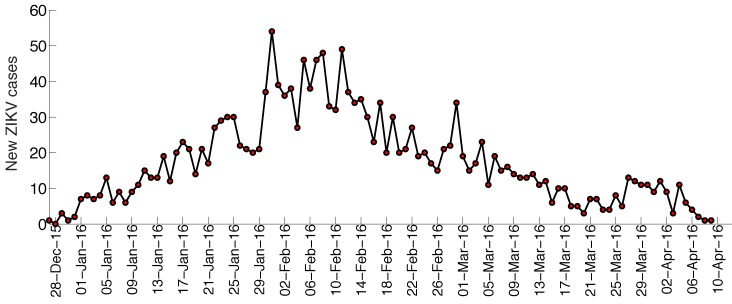
The time series for the number of new cases according to the date of symptoms onset of the Zika epidemic in Antioquia, Colombia.

**Reproduction number, gamma-distributed generation interval:**
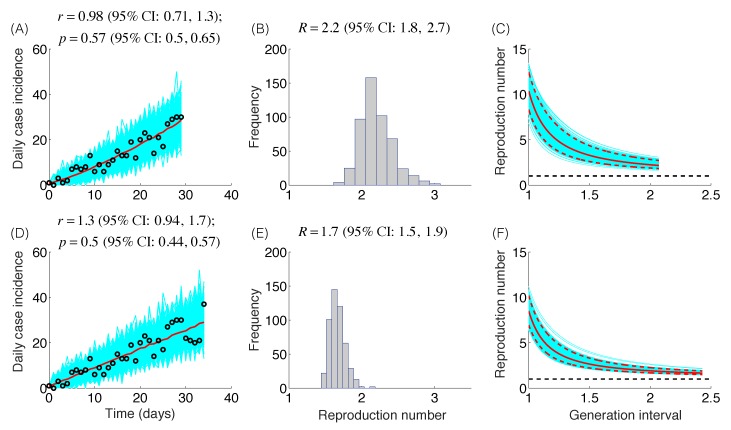
The model fits (A,D), empirical distributions of the reproduction number (B,E), and the estimated profiles of the reproduction number as a function of disease generations (C,F) using an increasing length of the early growth phase comprising 30 (A-C) and 35 (D-F) epidemic days. Model fit (red curve) and the associated uncertainty from individual bootstrapped curves assuming a Poisson error structure (cyan curves) to the case incidence data (black circles) are shown. Using 30 and 35 epidemic days of the Zika epidemic in Antioquia, the reproduction number was estimated at 2.2 (95%CI: 1.8, 2.7) and 1.7 (95% CI: 1.5, 1.9), respectively, given a gamma-distributed generation interval with the mean of 14 days (SD=2 days).

**Reproduction number, exponentially-distributed generation interval:**
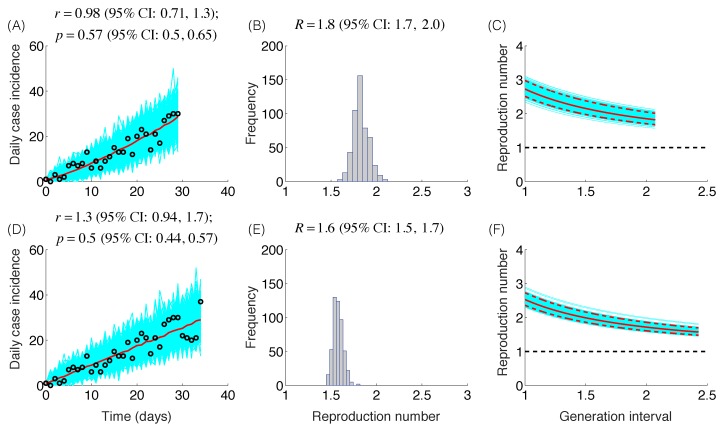
The model fits (A,D), empirical distributions of the reproduction number (B,E), and the estimated profiles of the reproduction number as a function of disease generations (C,F) using an increasing length of the early growth phase comprising 30 (A-C) and 35 (D-F) epidemic days. Model fit (red curve) and the associated uncertainty from individual bootstrapped curves assuming a Poisson error structure (cyan curves) to the case incidence data (black circles) are shown. Using 30 and 35 epidemic days of the Zika epidemic in Antioquia, the reproduction number was estimated at 1.8 (95%CI: 1.7, 2.0) and 1.6 (95% CI: 1.5, 1.7), respectively, given an exponentially-distributed generation interval with the mean of 14 days.

The GRM model provided reasonable forecasts of the expected epidemic size using incidence data for 20, 30, 40, and 50 days into the epidemic, with the sum of squared errors (SSE) decreasing from 35387 to 5256 with increasing the amount of epidemic data. In comparison, the sum of squared errors decreased from 45271 to 12248 for the logistic growth model (Figures 4-6). Uncertainty in the predicted epidemic final size was reduced with more data; Figure 6 shows mean prediction estimates of final epidemic size provided by the GRM which were within 9-22% of the targets for 30-40 days of epidemic data. By contrast, the logistic growth model based on early exponential growth dynamics consistently underestimated the epidemic size and was unable to provide a good fit to the early growth phase of the epidemic (Figures 5-6).

**Epidemic forecasts, GRM model:**
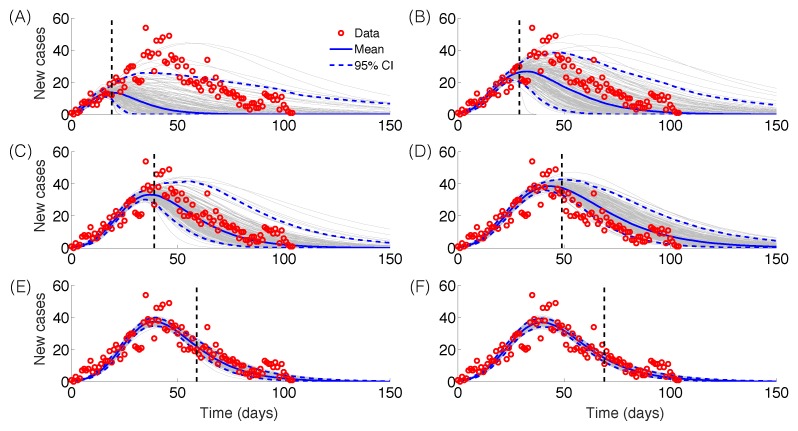
Epidemic forecasts based on the Generalized Richards Model (GRM) calibrated using an increasing amount of epidemic data (red circles): (A) 20, (B) 30, (C) 40, (D) 50, (E) 60 and (F) 70 epidemic days. The vertical dashed line indicates the end of the calibration period. The mean (solid blue line) and 95% CIs (dashed blue lines) of the model fit ensembles (gray curves) are shown.

**Epidemic forecasts, logistic growth model:**
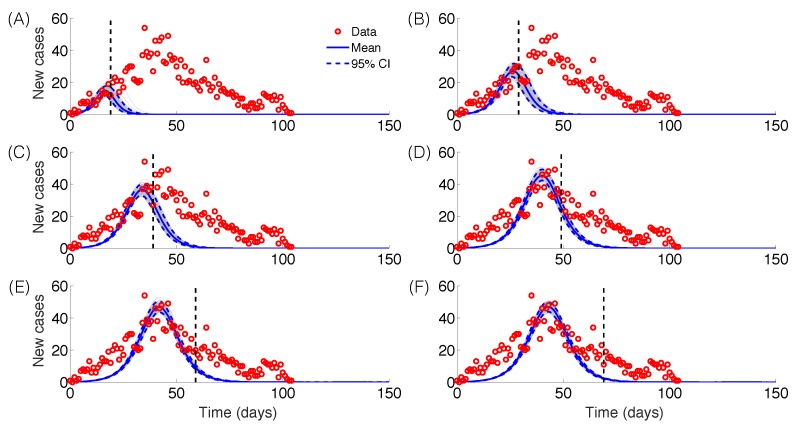
Epidemic forecasts based on the logistic growth model calibrated using an increasing amount of epidemic data (red circles): (A) 20, (B) 30, (C) 40, (D) 50, (E) 60 and (F) 70 epidemic days. The vertical dashed line indicates the end of the calibration period. The mean (solid blue line) and 95% CIs (dashed blue lines) of the model fit ensembles (gray curves) are shown.

**Predicting epidemic size, GRM model and logistic growth models:**
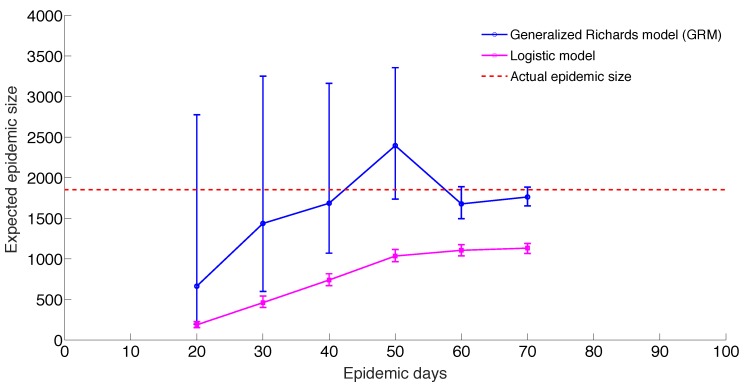
Mean and 95% CI of the forecasts for the expected epidemic final size of ZIKV cases in Antioquia, Colombia using the generalized Richards model (GRM) and the logistic growth model with increasing time-length of incidence data from 20 to 70 days.

Mean estimates of the deceleration of growth parameter (p) during the early growth phase derived by fitting the GGM model to increasing amounts of incidence data were relatively stable in the range p=0.44 – 0.65 (Figure 2), as shown in Figures 2-3. These estimates do not support an exponential epidemic growth profile, but indicate a sub-exponential growth profile with substantial uncertainty.

## Discussion

We applied simple phenomenological models applied to surveillance data of Zika cases from Antioquia, Colombia, to forecast the size of the Zika epidemic and evaluate the reproduction number within the first two disease generations. Using the GRM model that incorporates the possibility of sub-exponential growth dynamics^22^, we were able to generate reasonable forecasts of epidemic size within 22% of observed values based on 30 days of epidemic data. By contrast, the logistic growth model consistently underestimated the epidemic size and was not able to capture the early growth phase of the Zika epidemic, which exhibits an early sub-exponential growth phase (Figure 5-6). However, it is important to recall that the epidemic was of particularly short duration in this region of Colombia, as the peak was reached by day 36. It is possible that transmission was cut short by seasonal factors or control interventions. Our findings also underscore the need to characterize the temporal changes of the reproduction number, which can be substantial in some epidemics, as illustrated here with a drop from R~10 to R~2 within the first 28 days of the epidemic. Our results also emphasize the sensitivity of the reproduction number to assumptions about the shape of the generation interval distribution, in line with previous studies 28 ^,^ 29.

Prior studies have estimated the reproduction number assuming an exponential growth phase comprising ~ 2 generations of disease transmission for Zika outbreaks in the South Pacific (R ~ 1.8-5.8)^30^ and Colombia as a whole (R ~ 3.0-6.6)^31^. Our R estimates for Antioquia using a comparable length of the early growth phase as in ref. 31 are in the range 1.7-2.8, which are lower to those obtained from national weekly data for Colombia 31. It is likely that differences in model assumptions between^31^ and our work contributed significantly to differences in R estimates, together with differences in spatial resolution. In particular, the exponential growth assumption supports a sustained reproduction number during the early growth phase such that the number of secondary cases expected in the next disease generation grows proportionally to the number of cases in the current disease generation while sub-exponential (e.g., polynomial) growth implies a declining reproduction number even in the absence of interventions or susceptible depletion. The generalized-growth assumption used in our analyses allowed us to capture the growth kinetics directly from the daily series of case incidence, which is well characterized as it was based on dates of symptoms onset.

Compared to other methods for estimating the reproduction number (e.g., 28 ^,^ 32 ^,^ 33 ^,^ 34), our approach for estimating the reproduction number does not assume a particular profile of the early epidemic growth (e.g, exponential), but characterizes the epidemic growth phase using the GGM model via the deceleration of growth parameter p 23. A head-to-head comparison between methods should be the scope of future research using a combination of simulated and real data, and similar assumptions regarding serial interval and growth period.

The early growth profile of the Zika epidemic in Antioquia, Colombia, displayed sub-exponential growth dynamics where the deceleration of growth parameter, p, was estimated in the range 0.44 – 0.65. In the context of a highly susceptible population, it is likely that the spatial heterogeneity in the infection risk associated, for instance, with the presence of the relevant vector mosquito population could have contributed substantially to the observed polynomial growth profile. A variety of growth kinetics has been noted across a range of contemporary and historic outbreaks including influenza, Ebola, foot-and-mouth disease, HIV/AIDS, plague, measles and smallpox^22^. For instance, high values of p above 0.85, consistent with near-exponential growth, were estimated for a major plague epidemic in Bombay in 1905, the 1918 influenza pandemic in San Francisco, and a smallpox outbreak in Khulna, Bangladesh in 1972^35^. In contrast, p varied substantially in district-level Ebola epidemic outbreaks in West Africa, with an overall mean at ~0.6 consistent with polynomial growth^22^. The diversity of epidemic growth profiles observed in real epidemic outbreaks warrants further research focused on dissecting the mechanisms at play.

While we have compared the performance of the original Logistic growth model with that of the generalized Richards model in forecasting epidemic impact, a systematic comparison of performance, parameter correlations or parameter identifiability analyses across possible nested models (e.g., different model combinations with or without parameters \begin{equation*}p\end{equation*} and \begin{equation*}a\end{equation*} ) is outside the scope of this paper. Our goal here was to assess the ability of the enhanced Richards model incorporating flexible early growth profiles for forecasting short-term epidemic trajectory and epidemic size without attempting to interpret their collective parameter estimates across models or evaluate whether the actual parameter estimates are identifiable.

We used daily case series of suspected cases of Zika by date of symptoms onset captured by the surveillance system of Antioquia, Colombia. Hence, it is likely that this dataset only captures a small fraction of the true burden of Zika as a substantial fraction of the infections are asymptomatic and do not come to attention. As in other studies, our estimates of the reproduction number rely on surveillance data as a reliable proxy for the growth rate of Zika incidences.

While the epidemic in Antioquia has reached low incidence levels by mid-April 2016, the epidemic is still spreading in other departments of Colombia^36^. The particular reasons for the relative short duration of the epidemic in Antioquia are unclear, but is likely the result of interventions including mosquito control efforts and seasonal factors specific to the area. Overall the Zika epidemic in the Americas appears to be trending down, with most countries experiencing declining or stable weekly incidence rates. A comparison with the ZIKV epidemic in other countries is limited by the lack of refined spatial-temporal incidence data, or publicly available epidemic data^11^.

In summary, our study suggests that in the absence of reliable information about the transmission mechanisms of an emerging infection, simple phenomenological models can provide an early assessment of the potential scope of outbreaks in near real-time. Our study shows promising results for forecasting the temporal evolution of Zika epidemic in a province of Colombia; further work should extend this work to a broader geographic area. Further, phenomenological models cannot replace mechanistic models that incorporate mosquito dynamics, seasonality, different routes of transmission, and realistic distributions for epidemiological parameters. Such models are needed to predict the impact of intervention strategies against Zika and reduce the uncertainty of key epidemiological parameters such as the generation interval^24^. To the best of our knowledge, this is the first study reporting epidemic forecast of the Zika epidemic and estimates of the reproduction number based on daily case surveillance data by date of symptoms onset, and this approach could be readily tested in other settings.

## Competing Interests

The authors have declared that no competing interests exist.

## Data Availability Statement

The time series data are provided as Supporting Information.

## Supporting Information

S1 Data
